# Interdependence between dorsal and ventral hippocampus during spatial navigation

**DOI:** 10.1002/brb3.1410

**Published:** 2019-09-30

**Authors:** Shang Lin (Tommy) Lee, Dana Lew, Victoria Wickenheisser, Etan J. Markus

**Affiliations:** ^1^ Behavioral Neuroscience Division Department of Psychological Sciences University of Connecticut Storrs CT USA

**Keywords:** contralateral, dorsal hippocampus, inactivation, ipsilateral, spatial navigation, ventral hippocampus

## Abstract

**Introduction:**

The hippocampus is linked to the formation and retrieval of episodic memories and spatial navigation. In rats, it is an elongated structure divided into dorsal (septal) and ventral (temporal) regions paralleling the respective division in the posterior and anterior hippocampus in humans. The dorsal hippocampus has been suggested to be more important for spatial processing and the ventral to processing anxiety‐based behaviors. Far less is known regarding the degree to which these different regions interact during information processing. The anatomical connectivity suggests a flow of information between the dorsal and ventral regions; conversely, there are also commissural connections to the contralateral hippocampus. The current study examined the extent to which information from the dorsal hippocampus interacts with processing in the ipsilateral and contralateral ventral hippocampus following the acquisition of a spatial task.

**Methods:**

Rats were well‐trained on a spatial reference version of the water maze, followed by muscimol inactivation of different hippocampal subregions in a within‐animal repeated design. Various combinations of bilateral, ipsilateral, and contralateral infusions were used.

**Results:**

Combined dorsal and ventral inactivation produced a severe impairment in spatial performance. Inactivation of only the dorsal or ventral regions resulted in intermediate impairment with performance levels falling between controls and combined inactivation. Performance was impaired during contralateral inactivation and was almost equivalent to bilateral dorsal and ventral hippocampus inactivation, while ipsilateral inactivation resulted in little impairment.

**Conclusions:**

Taken together, results indicate that for spatial processing, the hippocampus functions as a single integrated structure along the longitudinal axis.

## Graphical abstract

The current study examined the extent to which information from the dorsal hippocampus interacts with processing in the ipsilateral and contralateral ventral hippocampus following the acquisition of a spatial task. Rats were well‐trained on a spatial reference version of the water maze, followed by muscimol inactivation of bilateral, ipsilateral, and contralateral hippocampal subregions in a within‐animal repeated design. Performance was impaired during contralateral inactivation and was almost equivalent to bilateral dorsal and ventral hippocampus inactivation, while ipsilateral inactivation resulted in little impairment. Taken together, results indicate that for spatial processing, the hippocampus functions as a single integrated structure along the longitudinal axis.

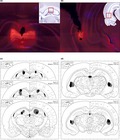

## 1 INTRODUCTION

In both humans and rodents, the hippocampus has long been linked to memory and spatial navigation (O'Keefe & Nadel, 1978). In rats, the hippocampus forms a long curved structure extending from near the medial septum down into the ventral temporal region of the brain. This longitudinal axis has received increased attention given finding of differences in connectivity and presumed function between the dorsal/septal (DH) and the ventral/temporal (VH) regions of the hippocampus.

### 1.1 Hippocampal inputs and outputs along the longitudinal axis

Cortical input to the hippocampus is led by the perforant path, from entorhinal cortex layer II to dentate gyrus and CA3, as well as entorhinal cortex layer III to CA1 (Witter et al., 2000). Each band of the entorhinal cortex projects topographically to a different septotemporal gradient of the dentate gyrus, with the most lateral band projecting to the dorsal dentate gyrus, and the medial band projecting to the ventral dentate gyrus (Dolorfo & Amaral, 1998).

The entorhinal cortex (EC) areas projecting predominately to DH include the dorsolateral EC and caudal MEC, which, respectively, receives major inputs from the perirhinal and postrhinal cortices. Conversely, VH receives predominate input from ventromedial EC, which receives major inputs from the piriform, infralimbic, and periamygdaloid cortices (Burwell & Amaral, 1998; Dolorfo & Amaral, 1998; Suzuki & Amaral, 1994; Witter & Groenewegen, 1984).

Output of the DH and subiculum primarily lead to the dorsal lateral septum, eventually to the mammillary body, processing memory and spatial navigation (Risold & Swanson, 1996). Hippocampus CA1 and subiculum are the primary sources of input returning to layer V of the entorhinal cortex (Witter, Doan, Jacobsen, Nilssen, & Ohara, 2017). Output of the VH projects to the medial amygdala (Kishi, Tsumori, Yokota, & Yasui, 2006; Pitkänen, Pikkarainen, Nurminen, & Ylinen, 2000), olfactory bulb (Van Groen & Wyss, 1990), ventral lateral septum, and subcortical structures associated with the hypothalamic–pituitary–adrenal axis (Risold & Swanson, 1996). Another topographical gradient involving the hippocampus includes its projection from DH to the lateral core of the nucleus accumbens versus VH to the medial shell of nucleus accumbens (Groenewegen, Zee, Kortschot, & Witter, 1987). These differences in inputs and outputs suggest the DH is primarily involved in spatial processing and VH for emotional/fear/olfactory learning (Fanselow & Dong, 2010).

### 1.2 The role of dorsal hippocampus in spatial processing

Early studies of hippocampal function involved the use of microelectrode recordings, which led to the discovery of "place cells" in the DH of freely moving rats (O'Keefe & Dostrovsky, 1971). Following this discovery, there have been numerous studies linking DH to spatial processing in water maze, radial maze, and contextual fear conditioning (Holt & Maren, 1999; Moser, Moser, & Andersen, 1993; Moser, Moser, Forrest, Andersen, & Morris, 1995; Pothuizen, Zhang, Jongen‐Rêlo, Feldon, & Yee, 2004). In adults with qualified training to become licensed taxi drivers, structural MRI revealed a selective increase in gray matter volume in posterior hippocampus, but not in the anterior region (Woollett & Maguire, 2011). Thus, there has been a long‐standing implication of dorsal/posterior hippocampus to spatial processing.

### 1.3 The role of ventral hippocampus in emotional/fear/olfactory processing

In contrast with the work on DH, some have suggested VH involvement in modulating anxiety‐related behavioral responses. These include auditory/contextual fear conditioning and defensive behaviors in the presence of cat odor/live cat (Bannerman et al., 2004; Bast, Zhang, & Feldon, 2001; Maren & Holt, 2004; Pentkowski, Blanchard, Lever, Litvin, & Blanchard, 2006; Richmond et al., 1999). Rats with VH lesions spent more time in the open arms of the elevated plus maze and the inner zone of the open field test (Kjelstrup et al., 2002; Weeden, Roberts, Kamm, & Kesner, 2015). Furthermore, they had reduced defecation and corticosterone secretion when exposed to a brightly lit test chamber (Kjelstrup et al., 2002). VH, but not DH, has also been shown to play an important role in working memory processing of odor information (Kesner, Hunsaker, & Ziegler, 2011; Weeden, Hu, Ho, & Kesner, 2014). This functional segregation is supported by anatomical differences found in VH projections to the olfactory bulb (Van Groen & Wyss, 1990). The functional dichotomy between dorsal (spatial) and ventral (emotional/olfactory) has been popular in prior literature; however, it is oversimplified.

### 1.4 The role of the ventral hippocampus in spatial processing

While there is much evidence to support a role for VH in emotional and odor processing, its role in spatial processing has been less clear. Some studies suggest there is no dependence of VH in spatial tasks (Bannerman et al., 2003, 1999; Moser et al., 1993; Pothuizen et al., 2004). However, unit‐recording studies found neurons in ventral CA1 and CA3 exhibit spatial tuning, thereby suggesting the area includes a mechanism involved in spatial memory (Jung, Wiener, & McNaughton, 1994; Kjelstrup et al., 2008; Komorowski et al., 2013). Importantly, “place cells” have also been found in human anterior hippocampus (Ekstrom et al., 2003). Hippocampal theta travels from septal to ventral poles (Lubenov & Siapas, 2009; Patel, Fujisawa, Berényi, Royer, & Buzsáki, 2012), and the synchrony in frequency may be a key in understanding functional integration within the hippocampus and its partner structures (Long, Bunce, & Chrobak, 2015).

The degree to which VH is necessary for spatial processing has been shown to be task and training dependent (Contreras, Pelc, Llofriu, Weitzenfeld, & Fellous, 2018; de Hoz, Knox, & Morris, 2003; de Hoz & Martin, 2014; Martin, de Hoz, & Morris, 2005; Richmond et al., 1999). Impairments in spatial memory performance were found after VH inactivation (Contreras et al., 2018; Floresco, Seamans, & Phillips, 1996; Loureiro et al., 2012; Wang & Cai, 2008). In addition, activation of the immediate early gene *Arc* was seen in both DH and VH during the retrieval of a recently acquired spatial memory (Beer, Chwiesko, & Sauvage, 2014; Gusev, Cui, Alkon, & Gubin, 2005). Therefore, the functional dichotomy between dorsal/ventral in spatial processing is not black and white.

### 1.5 The relationship between the dorsal and ventral hippocampus in processing information

Most studies address separately the role of DH or VH in processing spatial information, quantifying the degree of spatial impairment without one or both of the regions. It is important to note that intermediate hippocampus, with dorsal and ventral poles selectively lesioned, was found necessary and sufficient for rapid place learning (Bast, Wilson, Witter, & Morris, 2009). However, the hippocampus is a single continuous structure with anatomical connectivity that extends along the longitudinal axis. Thus, understanding the degree to which each region functions independently of the other is of prime interest.

Hippocampal CA3 cells contribute to associational projections across the long‐axis. Proximal CA3 preferentially projects in a septal direction, middle CA3 projects equally in septal and temporal directions, and distal CA3 projects in the ventral direction (Ishizuka, Weber, & Amaral, 1990). Projections from CA3 to CA1 follow the same organization, with CA3 neurons inside the hilus preferentially projecting to dorsal CA1, and distal CA3 neurons to ventral CA1 (Ishizuka et al., 1990; Li, Somogyi, Ylinen, & Buzsáki, 1994; Swanson, Wyss, & Cowan, 1978; Witter, 2007).

Within the hippocampus, CA3 pyramidal cells and dentate hilar mossy cells project to approximately two‐thirds of the septotemporal extent of the hippocampus (Amaral & Witter, 1989; Fricke & Cowan, 1978; Ishizuka et al., 1990; Li et al., 1994; Swanson et al., 1978). Despite ventral CA3 neurons having longer dendritic length compared with dorsal CA3 (Turner, Li, Pyapali, Ylinen, & Buzsaki, 1995), neurons in the temporal third in rats or the anterior extreme of the monkey hippocampus have relatively limited interconnections across the longitudinal axis (Kondo, Lavenex, & Amaral, 2008, 2009). These connections suggest a large degree of functional integration within the same hemisphere.

It has long been known that rodent's left and right hippocampi are connected (Blackstad, 1956). In rats, but not primates, dentate mossy cells project to the molecular layer of the contralateral dentate gyrus (Amaral, Scharfman, & Lavenex, 2007; Fricke & Cowan, 1978; Hjorth‐Simonsen & Laurberg, 1977; Laatsch & Cowan, 1967). CA3 cells project to both ipsilateral and contralateral sides (Gottlieb & Cowan, 1973; Laurberg, 1979; Swanson, Sawchenko, & Cowan, 1980). CA2 cells have commissural connections to contralateral CA1 (Shinohara et al., 2012), and to a lesser extent, CA1 cells have commissural projections to the contralateral CA1 (Andersen, Morris, Amaral, Bliss, & O'Keefe, 2007). These connections allow the transfer of information from the hippocampus in one hemisphere to the contralateral side.

While anatomical connectivity does not by itself indicate functional interactions, it provides a basis for such interactions. The current study was conducted to further examine the relative contributions of the DH and VH to spatial navigation, with a focus on the relationship between the two subregions. Rats were trained on a watermaze spatial reference memory task. After reaching asymptotic performance, different combinations of DH and VH were inactivated with muscimol in a within‐animal repeated design. Ipsilateral and contralateral infusions were used to determine the degree to which DH and VH were interdependent.

Inactivating the ipsilateral DH and VH in one hemisphere leaves the animal with one active hippocampus on the contralateral hemisphere. Contralateral inactivation of one DH and one VH leaves the same amount of hippocampal tissue active; however, the functional impact could vary. If the DH and VH act as independent modules, then contralateral inactivation should be equivalent to leaving one hippocampus active. Conversely, if the VH relies on input from its own DH, then a contralateral inactivation should be equivalent to bilaterally inactivation the entire hippocampus.

Unilateral damage to the hippocampus on one hemisphere has been shown to have little to no impact on spatial learning and retention (Czeh, Seress, Nadel, & Bures, 1998; Fenton, Arolfo, Nerad, & Bures, 1995; de Hoz, Moser, & Morris, 2005). Spatial learning was not shown to be lateralized in the left or right hemisphere of rats (Fenton et al., 1995; Port, Finamore, Noble, & Seybold, 2000). Interestingly, spatial learning was observed in rats with bilateral hippocampal lesions, leaving only 15% of their total hippocampus spared on one hemisphere (de Hoz et al., 2005). However, other studies have shown impairment in spatial reference and working memory following unilateral DH lesion (Port et al., 2000; Zou, Yamada, Sasa, & Nabeshima, 1999).

Based on the connectivity within the hippocampus, we hypothesized that the DH and VH regions are interdependent, and contralateral inactivation would cause greater impairments in spatial processing than unilateral inactivation.

## 2 MATERIALS AND METHODS

Six adult male Fisher‐344 rats (Harlan Laboratories), approximately 11 months old, were used in the present study. Rats were individually housed, with a 12‐hr light/dark cycle, and had ad libitum access to standard rat chow. These rats were previously tested in a radial arm watermaze in the same room using a different configuration of lights. While these animals had previous experience in a radial arm watermaze, all animals were overtrained on the current task before any drug infusions.

The watermaze was a circular plastic tub (140 cm diameter and 40 cm height) filled with water. A removable escape platform (10 cm diameter) constructed from clear Plexiglas was submerged 7.5 cm beneath the surface of the water.

**Figure 1:**
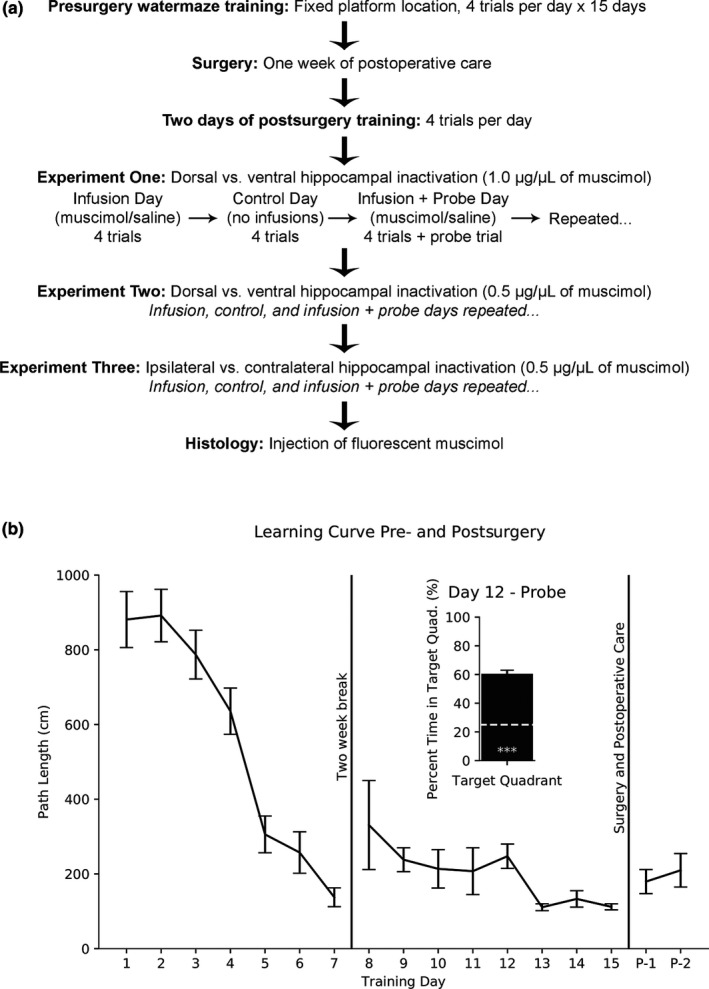
(a) Timeline of experimental procedures. Experimental days were repeated until each condition was given at least twice. (b) Swim path length (mean ± *SEM*) reached asymptotic levels across days. Inserted graph shows probe trial on Day 12 (mean ± *SEM*). One‐sample *t* test from 25% chance (white dashed line) is significant (*p* < .001***)

Presurgery (Figure 1a), rats were given four trials each day for seven days, followed by a two‐week break. The rats were trained again for eight days, for a total of 15 training days. There were four potential start locations in the maze. On a given day, rats were started from each of these positions in a pseudorandom order. The goal platform location was fixed for all experiments. Swim paths (X‐Y coordinates) were recorded using video‐tracking software (SMART, Pan Lab). The intertrial intervals were between 5 and 10 min apart.

Rats reached asymptotic performance by Training Day 13 (Figure 1b); a one‐way repeated measures ANOVA found no difference across Training Days 13–15. After reaching asymptotic performance, rats were anesthetized with 3.0% isoflurane in an induction chamber and then placed in a stereotaxic frame. The scalp was shaved, betadine was applied to the scalp, and ophthalmic ointment was applied to the eyes. Penicillin (0.1 ml) was injected subcutaneously to prevent infection. Meloxicam 0.1 mg/kg (Metacam; Boehringer Ingelheim) was injected subcutaneously to relieve pain. An incision was made to the scalp, and four small anchor screws were fastened to the skull. Based on physiological monitoring of respiration, heart rate, oxygen level, and reflexes, the isoflurane level was maintained between 1.0% and 3.0% throughout the entire surgery.

Guide cannulae (8IC315DCXXXC, Plastics ONE Inc.) were cut 7.0 mm below the pedestal, and dummy cannulae (8IC315GSPCLC, Plastics ONE Inc.) were cut to protrude 1.0 mm beyond the guide cannulae when securely fastened.

Bilateral cannulae implants aimed at the CA1 region of DH and VH. Cannulae aimed for DH had the guide and dummy cannulae tip pointing posteriorly at an angle of 20**°** in the sagittal plane. DH implants targeted the following coordinates: −2.8 AP, ±2.6 ML, and −4.2 DV relative to bregma, midline, and the skull surface in mm. The two remaining cannulae were vertically implanted into VH: −5.8 AP, ±5.3 ML, and −6.6 DV. Each rat had four cannulae implanted in total, and they were secured with dental cement.

After surgery, the rats were placed in a clean cage with a heating pad until ambulatory, after which they were single‐housed in a clean cage with bedding. Rats received Meloxicam 0.1 mg/kg subcutaneously for three days postsurgery. Animals were given one week of postoperative care before experimental testing.

The selective GABA_A_ receptor agonist muscimol was chosen because it leaves axonal conduction intact (McEown & Treit, 2010), an important factor for interpreting the results of contralateral inactivation. We used muscimol, in doses of 1.0 μg/μl (Experiment One) and 0.5 μg/μl (Experiment Two) dissolved in a vehicle solution of 0.9% saline (SAL). The same volume of 0.5 μl per injection site was used for both doses.

One hour prior to experimental testing, the guide and dummy cannulae were cleaned with 30% ethyl alcohol, and triple antibiotic ointment (Bacitracin Zinc, Neomycin Sulfate, and Polymyxin‐B Sulfate) was applied to the skin around the dental cement to protect against infection.

Thirty minutes prior to experimental testing, rats were restrained on the experimenter's lap, and a microinfusion pump (Genie Kent 1,000, Kent Scientific Corporation) was used to inject (0.166 μl/min) rats with 0.5 μl of muscimol or SAL per targeted cannulation site. The injector needle was left in the guide cannulae for one minute before the start of the infusion and one minute after the microinfusion pump stopped. All daily experiments were completed within two hours after drug administration. Based on prior literature of fluorescent muscimol drug spread (Allen et al., 2008), we expect a spread of 0.5–1.0 mm at each targeted injection site.

Experimental testing conditions consisted of infusion, control, and infusion plus probe days given on consecutive days. On infusion days, muscimol was used to temporarily inactivate specific regions of the hippocampus in half the rats, and the other half received SAL infusions. Rats were then tested in the watermaze for four trials. During control days, no infusions were administered, and rats were tested for four trials. Infusion plus probe days were similar to infusion days, but with an additional probe trial following four test trials. Experimental days continued until each condition was given at least twice. On scheduled infusion plus probe days, rats had a probe trial after the initial four trials of swimming to a hidden platform. During the probe trial, the platform was removed, and rats swam in the pool for 30 s.

Spatial performance was assessed based on swim path length, initial heading angle, probe target quadrant search time, and probe average distance from the platform. Decreased swim path length indicates better performance. Heading angle was defined as the angle between a direct route from the start location to the platform versus the actual route taken by the rat in the first three seconds. A smaller heading angle indicates better performance. If the platform was reached under three seconds, the heading angle was calculated as zero degrees.

During probe trials, the percent time spent in each quadrant was calculated. This variable provides a general index of search specificity (Morris, 1984). In addition to time spent, the average distance from the rat to the (removed) platform location was also calculated. This provides a more sensitive measure of search because the tracking data do not get binned into four categories (Vorhees & Williams, 2006).

The primary statistical analyses used for Experiments One and Two were one‐way ANOVA to determine differences between groups, followed by Tukey's post hoc tests to determine where differences occurred. Out of six rats, one rat had a single ventral cannula site that was unusable; therefore, we only included his bilateral DH data.

For Experiment Three, we collected ipsilateral and contralateral data from all rats; therefore, one‐way repeated measures ANOVA was used to determine differences. In addition, one‐sample *t* tests were used to compare groups to expected values.

**Figure 2:**
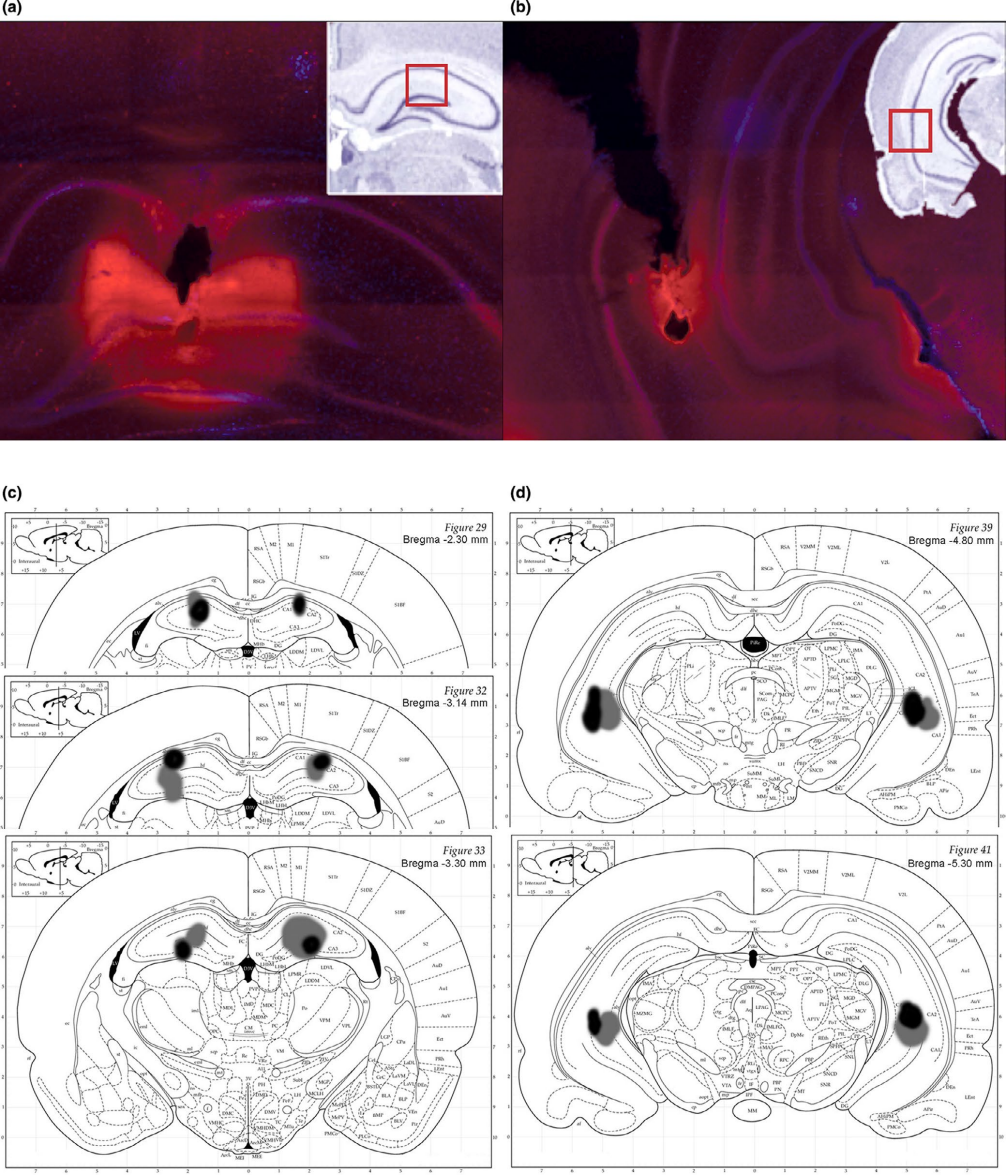
Histological verification of fluorescent muscimol spread in dorsal (a) and ventral (b) hippocampus. Insert shows the estimated location from the rat brain atlas (Paxinos & Watson, 2007). The largest (gray) and smallest (black) spread of fluorescent muscimol within the dorsal (c) and ventral (d) hippocampus, superimposed on their corresponding atlas sections

Upon completion of all experiments, rats were given an injection of fluorescent muscimol (Allen et al., 2008). The brain was sectioned in 50 μm increments using a vibratome, and ProLong® Gold Antifade Mountant with DAPI was used for histological verification. Cannulae placement and fluorescent drug spread were analyzed using a Zeiss Imager.M2 microscope, Orca‐R2 digital CCD camera, and Stereo Investigator software. Spread of fluorescent muscimol per injection site was identified for all animals (Figure 2).

## 3 RESULTS

### 3.1 Experiment One: DH versus VH inactivation with 1.0 μg/μl of muscimol

In the first experiment, 1.0 μg/μl concentration of muscimol was used to temporarily inactivate bilateral DH, VH, or combined DH + VH in well‐trained rats.

#### 3.1.1 No difference between saline conditions

**Figure 3:**
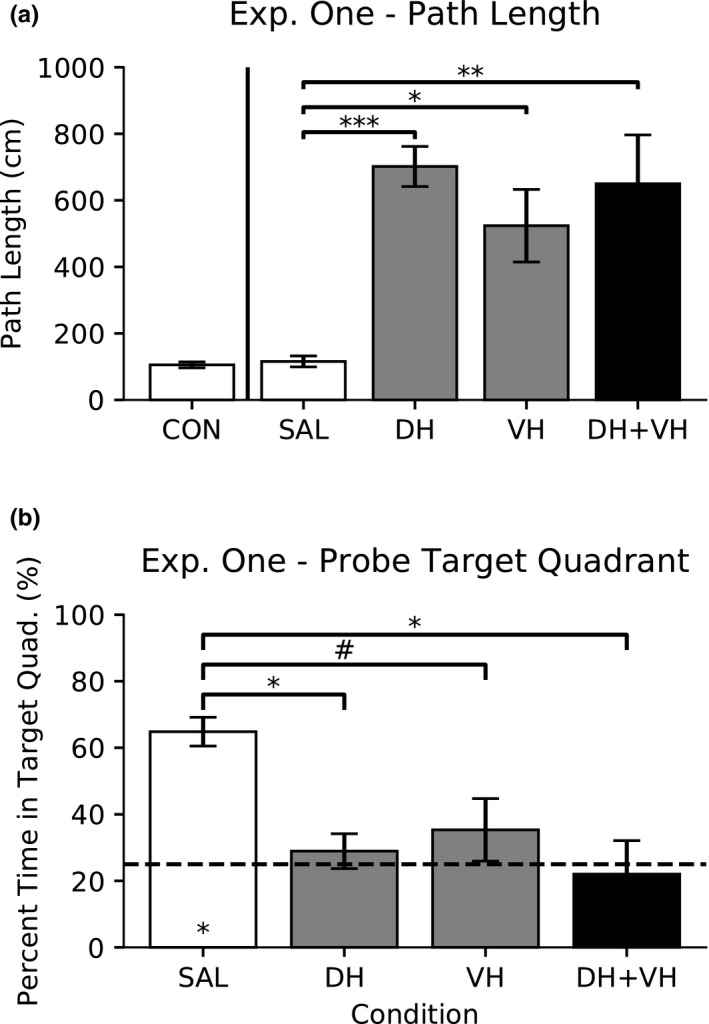
Experiment One: Dorsal versus ventral hippocampal inactivation with 1.0 μg/μl high dose of muscimol. Data are presented as mean ± *SEM*. (a) Swim path length. One‐way ANOVA, followed by Tukey's HSD post hoc comparisons, revealed differences in path length (*p* < .001***, *p* < .05*, and *p* < .01**). (b) Probe percent time spent in target quadrant. One‐way ANOVA, followed by Tukey's HSD post hoc comparisons, revealed differences in percent time in target quadrant (*p* < .05* and *p* = .099^#^). One‐sample *t* test versus 25% chance (dashed line) is significant for the saline condition (*p* < .05*)

A one‐way ANOVA was conducted to compare the effect of saline infusion in DH, VH, or DH + VH on swim path length. There was no significant difference, *F*(2,13) = 1.02, *p* > .10; therefore, the saline data were combined into one saline group (SAL, 115.76 cm ± 16.54 cm) to use as comparison with the muscimol conditions (Figure 3a).

A one‐way ANOVA was conducted to compare the effect of saline infusion in DH, VH, or DH + VH on the percent time spent in the target quadrant during probe trials. There was no significant difference, *F*(2,4) = 0.181, *p* > .10; therefore, the saline data were combined into one saline group (SAL, 64.83% ± 4.32%) to use as comparison with the muscimol conditions (Figure 3b).

#### 3.1.2 Path length

A one‐way ANOVA was conducted to compare the effect of saline and muscimol drug conditions on swim path length (Figure 3a). There was a significant difference, *F*(3,18) = 9.51, *p* < .001; therefore, post hoc comparisons were made using the Tukey's HSD test. Compared with saline (SAL), path length increased under 1.0 μg/μl of muscimol in DH (*p* < .001), VH (*p* < .01), and DH + VH (*p* < .05) inactivation. All other comparisons were not significant (*p* > .10).

#### 3.1.3 Probe percent time in target quadrant

A one‐way ANOVA was conducted to compare the effect of saline and muscimol drug conditions on the percent time spent in the target quadrant during probe trials (Figure 3b). There was a significant difference, *F*(3,8) = 6.02, *p* < .05; therefore, post hoc comparisons were made using the Tukey's HSD test. Compared with saline (SAL), percent time spent in the target quadrant during probe trials decreased under 1.0 μg/μl of muscimol in DH (*p* < .05) and DH + VH (*p* < .05) inactivation. The comparison between SAL and VH was trending (*p* = .099). All other comparisons were not significant (*p* > .10).

A one‐sample *t* test was conducted to determine whether the effect of saline or muscimol drug conditions on the percent time spent in the target quadrant during probe trials was different to expected chance percentage (25%). The SAL condition spent more percent time in the target quadrant during probe trials than expected chance (25%), *t*(2) = 9.22, *p* < .05. All other conditions were not different to chance (*p* > .10).

#### 3.1.4 Summary of Experiment One

Overall, rats who received a 1.0 μg/μl concentration of muscimol were severely impaired in their search for the hidden platform. This was true for all bilateral infusions, regardless of their location into DH, VH, or DH + VH. This ceiling effect would impede distinguishing between the effects of ipsilateral and contralateral infusions; therefore, the dose was reduced.

### 3.2 Experiment Two: DH versus VH inactivation with 0.5 μg/μl of muscimol

In the second experiment, 0.5 μg/μl concentration of muscimol was used to temporarily inactivate bilateral DH, VH, or combined DH + VH in well‐trained rats.

#### 3.2.1 No difference between saline conditions

**Figure 4:**
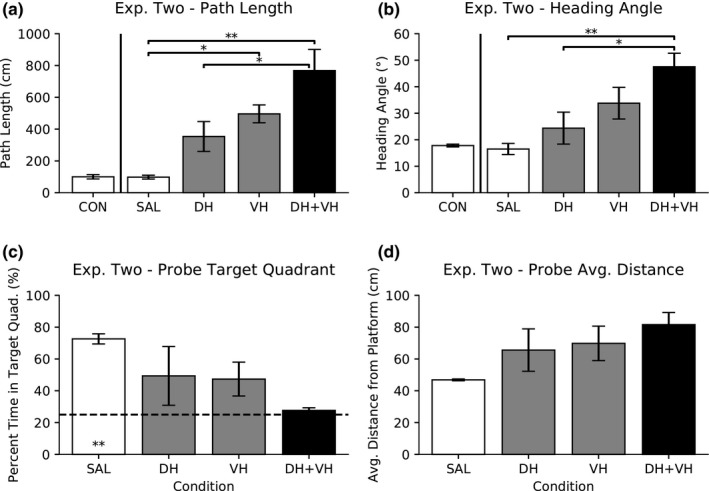
Experiment Two: Dorsal versus ventral hippocampal inactivation with 0.5 μg/μl low dose of muscimol. Data are presented as mean ± *SEM*. (a) Swim path length. One‐way ANOVA, followed by Tukey's HSD post hoc comparisons, revealed differences in path length (*p* < .05* and *p* < .01**). (b) Initial heading angle. One‐way ANOVA, followed by Tukey's HSD post hoc comparisons, revealed differences in initial heading angle (*p* < .05* and *p* < .01**). (c) Probe percent time spent in target quadrant. One‐way ANOVA was not found to be significant. One‐sample *t* test versus 25% chance (dashed line) is significant for the saline condition (*p* < .01**). (d) Probe average distance from platform. One‐way ANOVA was not found to be significant

A one‐way ANOVA was conducted to compare the effect of saline infusion in DH, VH, or DH + VH on swim path length. There was no significant difference, *F*(2,13) = 0.11, *p* > .10; therefore, the saline data were combined into one saline group (SAL, 98.07 cm ± 11.96 cm) to use as comparison with the muscimol conditions (Figure 4a).

A one‐way ANOVA was conducted to compare the effect of saline infusion in DH, VH, or DH + VH on initial heading angle. There was no significant difference, *F*(2,13) = 1.53, *p* > .10; therefore, the saline data were combined into one saline group (SAL, 16.50° ± 2.09°) to use as comparison with the muscimol conditions (Figure 4b).

A one‐way ANOVA was conducted to compare the effect of saline infusion in DH, VH, or DH + VH on the percent time spent in the target quadrant during probe trials. There was no significant difference, *F*(2,6) = 0.11, *p* > .10; therefore, the saline data were combined into one saline group (SAL, 72.65% ± 3.18%) to use as comparison with the muscimol conditions (Figure 4c).

A one‐way ANOVA was conducted to compare the effect of saline infusion in DH, VH, or DH + VH on the average distance from platform during probe trials. There was no significant difference, *F*(2,6) = 0.48, *p* > .10; therefore, the saline data were combined into one saline group (SAL, 46.86 cm ± 0.57 cm) to use as comparison with the muscimol conditions (Figure 4d).

#### 3.2.2 Path length

A one‐way ANOVA was conducted to compare the effect of saline and muscimol drug conditions on swim path length (Figure 4a). There was a significant difference, *F*(3,18) = 11.19, *p* < .001; therefore, post hoc comparisons were made using the Tukey's HSD test. Compared with saline (SAL), path length increased under 1.0 μg/μl of muscimol in VH (*p* < .05) and DH + VH (*p* < .01) inactivation. In addition, path length for DH + VH was larger than DH inactivation alone (*p* < .05). All other comparisons were not significant (*p* > .10).

#### 3.2.3 Heading angle

To better evaluate and compare spatial ability, in addition to swim path length, we also analyzed rat's initial heading angle three seconds after being placed in the watermaze. If the platform was reached in less than three seconds, the heading angle was calculated as zero degrees.

A one‐way ANOVA was conducted to compare the effect of saline and muscimol drug conditions on initial heading angle (Figure 4b). There was a significant difference, *F*(3,18) = 7.06, *p* < .01; therefore, post hoc comparisons were made using the Tukey's HSD test. Compared with saline (SAL), initial heading angle increased under 0.5 μg/μl of muscimol in DH + VH (*p* < .01) inactivation. In addition, initial heading angle for DH + VH was larger than DH inactivation alone (*p* < .05). All other comparisons were not significant (*p* > .10).

#### 3.2.4 Probe percent time in target quadrant

A one‐way ANOVA was conducted to compare the effect of saline and muscimol drug conditions on the percent time spent in the target quadrant during probe trials, and there was no significant difference, *F*(3,6) = 2.16, *p* > .10 (Figure 4c).

A one‐sample *t* test was conducted to determine whether the effect of saline or muscimol drug conditions on the percent time spent in the target quadrant during probe trials was different to expected chance percentage (25%). The SAL condition spent more percent time in the target quadrant during probe trials than expected chance (25%), *t*(2) = 14.99, *p* < .001. All other conditions were not different to chance (*p* > .10).

#### 3.2.5 Probe average distance from platform

To better evaluate and compare spatial ability, in addition to probe percent time in target quadrant, we also analyzed the average distance from where the platform was supposed to be during the 30 s probe trial.

A one‐way ANOVA was conducted to compare the effect of saline and muscimol drug conditions on the average distance from platform during probe trials, and there was no significant difference, *F*(3,6) = 2.22, *p* > .10 (Figure 4d).

#### 3.2.6 Summary of Experiment Two

Overall, rats who received a 0.5 μg/μl concentration of muscimol showed a differential impairment in their search the hidden platform. The combined DH + VH inactivation produced a severe impairment. Inactivation of only the DH or VH resulted in intermediate impairment, with performance levels falling between control and combined DH + VH inactivation. This intermediate impairment provided a range in which the effects of ipsilateral and contralateral infusions could be compared.

### 3.3 Experiment Three: Ipsilateral versus contralateral inactivation with 0.5 μg/μl of muscimol

In the third experiment, 0.5 μg/μl concentration of muscimol was used to temporarily inactivate ipsilateral (right hemisphere DH + right hemisphere VH) or contralateral (left hemisphere DH + right hemisphere VH) regions in well‐trained rats. If the DH and VH regions act as independent modules, then contralateral inactivation should be equivalent to leaving one hippocampus active, similar to an ipsilateral only inactivation. Conversely, if the VH region relies on input from the DH region, then a contralateral inactivation should be functionally equivalent to bilaterally inactivating DH + VH.

#### 3.3.1 No difference between saline conditions

**Figure 5:**
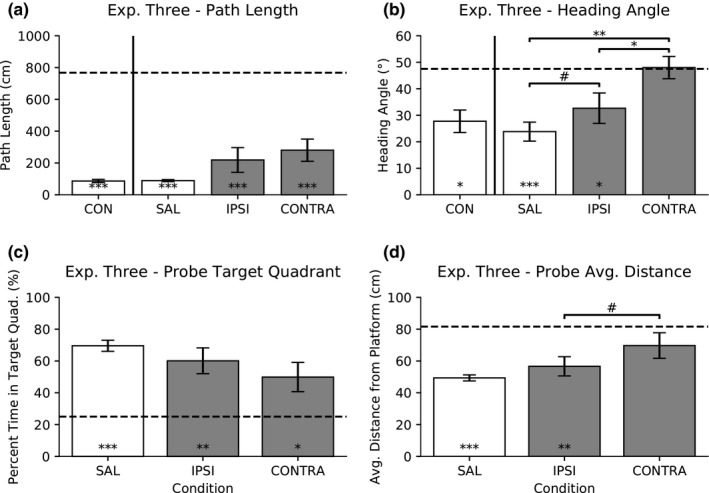
Experiment Three: IPSI versus CONTRA inactivation with 0.5 μg/μl low dose of muscimol. Data are presented as mean ± *SEM*. (a) Swim path length. One‐way repeated measures ANOVA was not found to be significant. One‐sample *t* test versus DH + VH from Experiment Two (dashed line) is significant for all conditions (*p* < .001***). (b) Initial heading angle. One‐way repeated ANOVA, followed by Tukey's HSD post hoc comparisons, revealed differences (*p* < .05*, *p* < .01**, and *p* = .096^#^). One‐sample *t* test versus DH + VH from Experiment Two (dashed line) is significant for saline and ipsilateral conditions (*p* < .001*** and *p* < .05* respectively). (c) Probe percent time spent in target quadrant. One‐way repeated measures ANOVA was not found to be significant. One‐sample *t* test versus DH + VH from Experiment Two (dashed line) is significant for the all conditions (*p* < .05*, *p* < .01**, and *p* < .001***). (d) Probe average distance from platform. One‐way repeated measures ANOVA, followed by Tukey's HSD post hoc comparisons, revealed a trend (*p* = .09). One‐sample *t* test versus DH + VH from Experiment Two (dashed line) is significant for SAL and IPSI conditions (*p* < .001*** and *p* < .01**, respectively)

A paired *t* test was conducted to compare the effect of saline infusion in ipsilateral (IPSI) or contralateral (CONTRA) regions on swim path length. There was no significant difference, *t*(5) = 1.97, *p* > .10; therefore, the saline data were combined into one saline group (SAL, 89.0 cm ± 6.27 cm) to use as comparison with the muscimol conditions (Figure 5a).

A paired *t* test was conducted to compare the effect of saline infusion in ipsilateral (IPSI) or contralateral (CONTRA) regions on initial heading angle. There was no significant difference, *t*(5) = −0.96, *p* > .10; therefore, the saline data were combined into one saline group (SAL, 23.83° ± 3.59°) to use as comparison with the muscimol conditions (Figure 5b).

A paired *t* test was conducted to compare the effect of saline infusion in ipsilateral (IPSI) or contralateral (CONTRA) regions on the percent time spent in the target quadrant during probe trials. There was no significant difference, *t*(5) = −1.09, *p* > .10; therefore, the saline data were combined into one saline group (SAL, 69.56% ± 3.48%) to use as comparison with the muscimol conditions (Figure 5c).

A paired *t* test was conducted to compare the effect of saline infusion in ipsilateral (IPSI) or contralateral (CONTRA) regions on the average distance from platform during probe trials. There was no significant difference, *t*(5) = 1.24, *p* > .10; therefore, the saline data were combined into one saline group (SAL, 49.35 cm ± 1.89 cm) to use as comparison with the muscimol conditions (Figure 5d).

#### 3.3.2 Path length

A one‐way repeated measures ANOVA was conducted to compare the effect of saline and muscimol drug conditions on swim path length, and there was no significant difference, *F*(2,10) = 3.60, *p* = .07 (Figure 5a).

A one‐sample *t* test was conducted to determine whether the effect of saline or muscimol drug conditions on the swim path length was different to the combined DH + VH results from Experiment Two (767.88 cm). The CON (*t*(5) = −69.43, *p* < .001), SAL (*t*(5) = −108.27, *p* < .001), IPSI (*t*(5) = −7.06, *p* < .001), and CONTRA (*t*(5) = −6.99, *p* < .001) conditions all resulted in a shorter path length than the combined DH + VH inactivation from Experiment Two.

A one‐way repeated measures ANOVA was conducted to determine whether the path length decreased as the rats progress from Experiments One, Two, and Three. There was no significant difference between control groups across experiments, *F*(2,10) = 1.61, *p* > .10. In addition, there was no significant difference between saline groups across experiments, *F*(2,10) = 2.57, *p* > .10. Repeated infusions did not change the asymptotic control or saline performance.

#### 3.3.3 Heading angle

A one‐way repeated measures ANOVA was conducted to compare the effect of saline and muscimol drug conditions on initial heading angle (Figure 5b). There was a significant difference, *F*(2,10) = 19.29, *p* < .001; therefore, post hoc comparisons were made using the Tukey's HSD test. Compared with saline (SAL), initial heading angle increased under 0.5 μg/μl of muscimol in CONTRA inactivation (*p* < .01). The comparison between SAL and IPSI was trending (*p* = .096). Lastly, CONTRA inactivation produced a larger initial heading angle than IPSI inactivation (*p* < .05).

A one‐sample *t* test was conducted to determine whether the effect of saline or muscimol drug conditions on initial heading angle was different to the combined DH + VH results from Experiment Two (47.5°). The CON (*t*(5) = −2.66, *p* < .05), SAL (*t*(5) = −6.60, *p* < .001), and IPSI (*t*(5) = −2.59, *p* < .05) conditions resulted in a smaller initial heading angle than the combined DH + VH inactivation from Experiment Two. There was no difference in initial heading angle between CONTRA and the combined DH + VH inactivation from Experiment Two (*t*(5) = 0.11, *p* > .10).

#### 3.3.4 Probe percent time in target quadrant

A one‐way repeated measures ANOVA was conducted to compare the effect of saline and muscimol drug conditions on the percent time spent in the target quadrant during probe trials (Figure 5c). There was a significant difference, *F*(2,10) = 4.13, *p* < .05; therefore, post hoc comparisons were made using the Tukey's HSD test. All post hoc comparisons were not significant (*p* > .10).

A one‐sample *t* test was conducted to determine whether the effect of saline or muscimol drug conditions on the percent time spent in the target quadrant during probe trials was different to expected chance percentage (25%). The SAL (*t*(5) = 12.82, *p* < .001), IPSI (*t*(5) = 4.33, *p* < .01), and CONTRA (*t*(5) = 2.71, *p* < .05) conditions all spent more percent time in the target quadrant during probe trials than expected chance percentage.

#### 3.3.5 Probe average distance from platform

A one‐way repeated measures ANOVA was conducted to compare the effect of saline and muscimol drug conditions on the average distance from platform during probe trials (Figure 5d). There was a significant difference, *F*(2,10) = 5.46, *p* < .05; therefore, post hoc comparisons were made using the Tukey's HSD test. Compared with IPSI, the comparison of average distance from platform during probe trials in CONTRA inactivation was trending (*p* = .09). All other comparisons were not significant (*p* > .10).

A one‐sample *t* test was conducted to determine whether the effect of saline or muscimol drug conditions on the average distance from platform during probe trials was different to the combined DH + VH results from Experiment Two (81.6 cm). The SAL (*t*(5) = −17.06, *p* < .001) and IPSI (*t*(5) = −4.14, *p* < .01) conditions produced a smaller average distance from platform during probe trials than the combined DH + VH inactivation from Experiment Two. There was no difference in average distance from platform during probe trials between CONTRA and the combined DH + VH inactivation from Experiment Two (*t*(5) = −1.48, *p* > .10).

## 4 DISCUSSION

The current study was conducted to determine the interdependence between dorsal and ventral subregions of the hippocampus in spatial processing of a well‐learned task and familiar room. Rats were trained on a spatial reference memory watermaze task until asymptotic performance. Following training, different combinations of dorsal and ventral subregions were temporarily inactivated with muscimol in a within‐animal repeated design. Ipsilateral and contralateral infusions were used to determine the degree to which the dorsal and ventral regions were independent.

Before determining interdependence between the dorsal and ventral regions, the effects of bilateral inactivation were conducted. A high dose of muscimol (1.0 μg/μl), equivalent to 4.4 nmol, severely impaired performance. The effects of DH inactivation alone or VH inactivation alone were similar to a combined DH + VH bilateral inactivation (Experiment One).

Memory impairments as a result of muscimol infusion have been shown to be dose‐dependent. Muscimol infusion into the medial septum with a dose of 3.0 or 1.5 nmol, but not 0.75 nmol, produced impairments in a radial arm maze task (Chrobak, Stackman, & Walsh, 1989). In the hippocampus, a dose as low as 0.26 nmol shifted rats from a place learning strategy to a response strategy (McElroy & Korol, 2005). We reduced the dose from 1.0 μg/μl in Experiment One to 0.5 μg/μl in Experiment Two and used the same injection volume of 0.5 μg/μl across both experiments.

In the current study, a lower dose of muscimol (0.5 μg/μl), equivalent to 2.2 nmol, resulted in a more graded level of impairment (Experiment Two). Overall, bilateral infusions restricted to one subregion were less disruptive than combined DH + VH bilateral inactivation. Notably, VH inactivation had a similar impact on spatial performance as DH inactivation, indicating a role for the VH in our task. Given prior literature, we would have expected DH inactivation to produce a greater impairment than VH inactivation (Moser et al., 1993; M. B. Moser et al., 1995). However, these differences are highly dependent on the training protocol implemented (Contreras et al., 2018; de Hoz et al., 2003; de Hoz & Martin, 2014; Martin et al., 2005; Richmond et al., 1999).

To assess the degree of interdependence between different subregions within the hippocampus, ipsilateral and contralateral inactivation of the different subregions was applied. This disconnection approach has been used previously to examine the hemispheric interdependence of spatial processing between the medial prefrontal cortex and DH (Churchwell & Kesner, 2011), the prelimbic cortex and VH (Wang & Cai, 2008), and the lateral septum and VH (Trent & Menard, 2010).

Inactivating the dorsal and ventral hippocampus in one hemisphere leaves the animal with one active hippocampus on the contralateral side. If the dorsal and ventral regions act as completely independent modules, then contralateral inactivation should be equivalent to leaving one hippocampus active. Conversely, if the ventral region relies on input from its own dorsal region, then a contralateral inactivation should be functionally equivalent to inactivating the DH + VH in both hemispheres.

Performance was severely impaired during contralateral inactivation, almost equivalent to combined DH + VH inactivation, while ipsilateral inactivation resulted in little impairment. Initial heading angle under contralateral inactivation was larger than saline and ipsilateral conditions, but ipsilateral inactivation was not larger than the saline condition.

The effects of contralateral inactivation were similar to a combined DH + VH inactivation on multiple measures of spatial performance. Ipsilateral inactivation had a smaller initial heading angle than combined DH + VH inactivation; however, contralateral inactivation was not different from combined DH + VH inactivation. In addition, ipsilateral inactivation had a smaller probe average distance from platform than combined DH + VH inactivation; however, contralateral inactivation was not different from combined DH + VH inactivation. Taken together, these data indicate that the ventral regions are dependent upon ipsilateral input from the dorsal regions in our spatial task.

Notably, the rats in the current study were well‐trained prior to muscimol inactivation, so the results are relevant to spatial orientation in the environment and expression of a learned spatial task. It would be interesting to determine the degree to which dorsal and ventral hippocampal subregions are interdependent during task acquisition. Presumably, during acquisition there may be an even greater degree of hippocampal integration. This assumption is supported by the finding that coherence between the dorsal and ventral subregions increase when the rat is confronted with a spatial working memory task (Schmidt et al., 2013). Furthermore, it was recently shown that both DH and VH are important for spatial navigation within increasing environmental complexities (Contreras et al., 2018).

Overall, contralateral inactivation caused a greater impairment than ipsilateral inactivation; however, it did not always equal combined DH + VH inactivation. On general measures of spatial selectivity, such as path length and probe percent time in target quadrant, rats with contralateral inactivation continued to show spatial selectivity. However, using more precise measures of spatial selectivity, such as initial heading angle and probe average distance from platform, manifested a contralateral inactivation impairment similar to the combined DH + VH inactivation. One reason why the contralateral inactivation was somewhat less disruptive than combined DH + VH inactivation may be attributed to the widespread commissural connections across hemispheres (Amaral et al., 2007; Blackstad, 1956; Gottlieb & Cowan, 1973; Hjorth‐Simonsen & Laurberg, 1977; Swanson et al., 1980). Thus, commissural connections across hemispheres could provide some general spatial data; however, this did not allow for precise spatial selectivity.

In summary, the behavioral results suggest that the flow of information along the dorsoventral axis is an important component of normal hippocampal function. This conclusion is in agreement with the anatomical circuitry showing projections from the dorsal CA3 and dentate gyrus to the more ventral part of the hippocampus (Amaral & Witter, 1989; Ishizuka et al., 1990; Swanson et al., 1978). Conversely, the data suggest only a weak impact of commissural inputs on hippocampal processing. Taken together, the data indicate spatial processing in the hippocampus functions as a single integrated structure along the longitudinal axis.

## CONFLICT OF INTEREST

No conflicts of interest, financial or otherwise, are declared by the authors.

## Data Availability

The data that support the findings of this study are available from the corresponding author upon reasonable request.

## References

[brb31410-bib-0001] Allen, T. A. , Narayanan, N. S. , Kholodar‐Smith, D. B. , Zhao, Y. , Laubach, M. , & Brown, T. H. (2008). Imaging the spread of reversible brain inactivations using fluorescent muscimol. Journal of Neuroscience Methods, 171(1), 30–38. 10.1016/j.jneumeth.2008.01.033 18377997 PMC2440580

[brb31410-bib-0002] Amaral, D. G. , Scharfman, H. E. , & Lavenex, P. (2007). The dentate gyrus: Fundamental neuroanatomical organization (dentate gyrus for dummies). Progress in Brain Research, 163, 3–790. 10.1016/S0079-6123(07)63001-5 17765709 PMC2492885

[brb31410-bib-0003] Amaral, D. G. , & Witter, M. P. (1989). The three‐dimensional organization of the hippocampal formation: A review of anatomical data. Neuroscience, 31(3), 571–591. 10.1016/0306-4522(89)90424-7 2687721

[brb31410-bib-0004] Andersen, P. , Morris, R. , Amaral, D. , Bliss, T. , & O'Keefe, J. (2007). The hippocampus book. Oxford, UK: Oxford University Press Inc.

[brb31410-bib-0005] Bannerman, D. M. , Grubb, M. , Deacon, R. M. , Yee, B. , Feldon, J. , & Rawlins, J. N. (2003). Ventral hippocampal lesions affect anxiety but not spatial learning. Behavioural Brain Research, 139(1–2), 197–213. 10.1016/S0166-4328(02)00268-1 12642189

[brb31410-bib-0006] Bannerman, D. M. , Rawlins, J. , McHugh, S. B. , Deacon, R. , Yee, B. K. , Bast, T. , … Feldon, J. (2004). Regional dissociations within the hippocampus—memory and anxiety. Neuroscience & Biobehavioral Reviews, 28(3), 273–283. 10.1016/j.neubiorev.2004.03.004 15225971

[brb31410-bib-0007] Bannerman, D. M. , Yee, B. K. , Good, M. A. , Heupel, M. J. , Iversen, S. D. , & Rawlins, J. N. (1999). Double dissociation of function within the hippocampus: A comparison of dorsal, ventral, and complete hippocampal cytotoxic lesions. Behavioral Neuroscience, 113(6), 1170–1188. 10.1037/0735-7044.113.6.1170 10636297

[brb31410-bib-0008] Bast, T. , Wilson, I. A. , Witter, M. P. , & Morris, R. G. M. (2009). From rapid place learning to behavioral performance: A key role for the intermediate hippocampus. PLoS Biology, 7(4), 730–746. 10.1371/journal.pbio.1000089 PMC2671558 19385719

[brb31410-bib-0009] Bast, T. , Zhang, W.‐N. , & Feldon, J. (2001). The ventral hippocampus and fear conditioning in rats. Experimental Brain Research, 139(1), 39–52. 10.1007/s002210100746 11482842

[brb31410-bib-0010] Beer, Z. , Chwiesko, C. , & Sauvage, M. M. (2014). Processing of spatial and non‐spatial information reveals functional homogeneity along the dorso‐ventral axis of CA3, but not CA1. Neurobiology of Learning and Memory, 111, 56–64. 10.1016/j.nlm.2014.03.001 24657342

[brb31410-bib-0011] Blackstad, T. W. (1956). Commissural connections of the hippocampal region in the rat, with special reference to their mode of termination. The Journal of Comparative Neurology, 105(3), 417–537. 10.1002/cne.901050305 13385382

[brb31410-bib-0012] Burwell, R. D. , & Amaral, D. G. (1998). Perirhinal and postrhinal cortices of the rat: Interconnectivity and connections with the entorhinal cortex. The Journal of Comparative Neurology, 391(3), 293–321. 10.1002/(SICI)1096-9861(19980216)391:3<293:AID-CNE2>3.0.CO;2-X 9492202

[brb31410-bib-0013] Chrobak, J. J. , Stackman, R. W. , & Walsh, T. J. (1989). Intraseptal administration of muscimol produces dose‐dependent memory impairments in the rat. Behavioral and Neural Biology, 52(3), 357–369. 10.1016/S0163-1047(89)90472-X 2556105

[brb31410-bib-0014] Churchwell, J. C. , & Kesner, R. P. (2011). Hippocampal‐prefrontal dynamics in spatial working memory: Interactions and independent parallel processing. Behavioural Brain Research, 225(2), 389–395. 10.1016/j.bbr.2011.07.045 21839780 PMC3586941

[brb31410-bib-0015] Contreras, M. , Pelc, T. , Llofriu, M. , Weitzenfeld, A. , & Fellous, J.‐M. (2018). The ventral hippocampus is involved in multi‐goal obstacle‐rich spatial navigation. Hippocampus, 28(12), 853–866. 10.1002/hipo.22993 30067283

[brb31410-bib-0016] Czeh, B. , Seress, L. , Nadel, L. , & Bures, J. (1998). Lateralized fascia dentata lesion and blockade of one hippocampus: Effect on spatial memory in rats. Hippocampus, 8(6), 647–650. 10.1002/(SICI)1098-1063(1998)8:6<647:AID-HIPO7>3.0.CO;2-L 9882022

[brb31410-bib-0017] de Hoz, L. , Knox, J. , & Morris, R. G. M. (2003). Longitudinal axis of the hippocampus: Both septal and temporal poles of the hippocampus support water maze spatial learning depending on the training protocol. Hippocampus, 13(5), 587–603. 10.1002/hipo.10079 12921349

[brb31410-bib-0018] de Hoz, L. , & Martin, S. J. (2014). Double dissociation between the contributions of the septal and temporal hippocampus to spatial learning: The role of prior experience. Hippocampus, 24(8), 990–1005. 10.1002/hipo.22285 24753035

[brb31410-bib-0019] de Hoz, L. , Moser, E. I. , & Morris, R. G. M. (2005). Spatial learning with unilateral and bilateral hippocampal networks. European Journal of Neuroscience, 22(3), 745–754. 10.1111/j.1460-9568.2005.04255.x 16101756

[brb31410-bib-0020] Dolorfo, C. L. , & Amaral, D. G. (1998). Entorhinal cortex of the rat: Topographic organization of the cells of origin of the perforant path projection to the dentate gyrus. The Journal of Comparative Neurology, 398(1), 25–48. 10.1002/(SICI)1096-9861(19980817)398:1<25:AID-CNE3>3.0.CO;2-B 9703026

[brb31410-bib-0021] Ekstrom, A. D. , Kahana, M. J. , Caplan, J. B. , Fields, T. A. , Isham, E. A. , Newman, E. L. , & Fried, I. (2003). Cellular networks underlying human spatial navigation. Nature, 425(6954), 184–188. 10.1038/nature01964 12968182

[brb31410-bib-0022] Fanselow, M. S. , & Dong, H.‐W. (2010). Are the dorsal and ventral hippocampus functionally distinct structures? Neuron, 65(1), 7–19. 10.1016/j.neuron.2009.11.031 20152109 PMC2822727

[brb31410-bib-0023] Fenton, A. A. , Arolfo, M. P. , Nerad, L. , & Bures, J. (1995). Interhippocampal synthesis of lateralized place navigation engrams. Hippocampus, 5(1), 16–24. 10.1002/hipo.450050104 7787943

[brb31410-bib-0024] Floresco, S. B. , Seamans, J. K. , & Phillips, A. G. (1996). Differential effects of lidocaine infusions into the ventral CA1/subiculum or the nucleus accumbens on the acquisition and retention of spatial information. Behavioural Brain Research, 81, 163–171. 10.1016/S0166-4328(96)00058-7 8950013

[brb31410-bib-0025] Fricke, R. , & Cowan, W. M. (1978). An autoradiographic study of the commissural and ipsilateral hippocampo‐dentate projections in the adult rat. The Journal of Comparative Neurology, 181(2), 253–269. 10.1002/cne.901810204 567658

[brb31410-bib-0026] Gottlieb, D. I. , & Cowan, W. M. (1973). Autoradiographic studies of the commissural and ipsilateral association connections of the hippocampus and dentate gyrus. I. The commissural connections. The Journal of Comparative Neurology, 149(4), 393–421. 10.1002/cne.901490402 4715298

[brb31410-bib-0027] Groenewegen, H. J. , der Zee, E.‐V.‐V. , te Kortschot, A. , & Witter, M. P. (1987). Organization of the projections from the subiculum to the ventral striatum in the rat. A study using anterograde transport of Phaseolus vulgaris leucoagglutinin. Neuroscience, 23(1), 103–120. 10.1016/0306-4522(87)90275-2 3683859

[brb31410-bib-0028] Gusev, P. A. , Cui, C. , Alkon, D. L. , & Gubin, A. N. (2005). Topography of Arc/Arg3.1 mRNA expression in the dorsal and ventral hippocampus induced by recent and remote spatial memory recall: Dissociation of CA3 and CA1 activation. The Journal of Neuroscience, 25(41), 9384–9397. 10.1523/JNEUROSCI.0832-05.2005 16221847 PMC6725713

[brb31410-bib-0029] Hjorth‐Simonsen, A. , & Laurberg, S. (1977). Commissural connections of the dentate area in the rat. The Journal of Comparative Neurology, 174(4), 591–605. 10.1002/cne.901740404 903420

[brb31410-bib-0030] Holt, W. , & Maren, S. (1999). Muscimol Inactivation of the Dorsal Hippocampus Impairs Contextual Retrieval of Fear Memory. The Journal of Neuroscience, 19(20), 9054–9062. 10.1523/JNEUROSCI.19-20-09054.1999 10516322 PMC6782751

[brb31410-bib-0031] Ishizuka, N. , Weber, J. , & Amaral, D. G. (1990). Organization of intrahippocampal projections originating from CA3 pyramidal cells in the rat. The Journal of Comparative Neurology, 295(4), 580–623. 10.1002/cne.902950407 2358523

[brb31410-bib-0032] Jung, M. , Wiener, S. , & McNaughton, B. (1994). Comparison of spatial firing characteristics of units in dorsal and ventral hippocampus of the rat. The Journal of Neuroscience, 14(12), 7347–7356. 10.1523/JNEUROSCI.14-12-07347.1994 7996180 PMC6576902

[brb31410-bib-0033] Kesner, R. P. , Hunsaker, M. R. , & Ziegler, W. (2011). The role of the dorsal and ventral hippocampus in olfactory working memory. Neurobiology of Learning and Memory, 96(2), 361–366. 10.1016/j.nlm.2011.06.011 21742047

[brb31410-bib-0034] Kishi, T. , Tsumori, T. , Yokota, S. , & Yasui, Y. (2006). Topographical projection from the hippocampal formation to the amygdala: A combined anterograde and retrograde tracing study in the rat. Journal of Comparative Neurology, 496, 349–368. 10.1002/cne.20919 16566004

[brb31410-bib-0035] Kjelstrup, K. B. , Solstad, T. , Brun, V. H. , Hafting, T. , Leutgeb, S. , Witter, M. P. , … Moser, M.‐B. (2008). Finite Scale of Spatial Representation in the Hippocampus. Science, 321(5885), 140–143. 10.1126/science.1157086 18599792

[brb31410-bib-0036] Kjelstrup, K. , Tuvnes, F. A. , Steffenach, H.‐A. , Murison, R. , Moser, E. I. , & Moser, M.‐B. (2002). Reduced fear expression after lesions of the ventral hippocampus. Proceedings of the National Academy of Sciences of the United States of America, 99, 10825–10830. 10.1073/pnas.152112399 12149439 PMC125057

[brb31410-bib-0037] Komorowski, R. W. , Garcia, C. G. , Wilson, A. , Hattori, S. , Howard, M. W. , & Eichenbaum, H. (2013). Ventral hippocampal neurons are shaped by experience to represent behaviorally relevant contexts. Journal of Neuroscience, 33(18), 8079–8087. 10.1523/JNEUROSCI.5458-12.2013 23637197 PMC3667351

[brb31410-bib-0038] Kondo, H. , Lavenex, P. , & Amaral, D. G. (2008). Intrinsic connections of the macaque monkey hippocampal formation: I. Dentate gyrus. The Journal of Comparative Neurology, 511(4), 497–520. 10.1002/cne.21825 18844234 PMC2597032

[brb31410-bib-0039] Kondo, H. , Lavenex, P. , & Amaral, D. G. (2009). Intrinsic connections of the macaque monkey hippocampal formation: II. CA3 connections. The Journal of Comparative Neurology, 515(3), 349–377. 10.1002/cne.22056 19425110 PMC4386899

[brb31410-bib-0040] Laatsch, R. H. , & Cowan, W. M. (1967). Electron microscopic studies of the dentate gyrus of the rat. II. Degeneration of commissural afferents. The Journal of Comparative Neurology, 130(3), 241–261. 10.1002/cne.901300306 5971657

[brb31410-bib-0041] Laurberg, S. (1979). Commissural and intrinsic connections of the rat hippocampus. The Journal of Comparative Neurology, 184(4), 685–708. 10.1002/cne.901840405 422759

[brb31410-bib-0042] Li, X.‐G. , Somogyi, P. , Ylinen, A. , & Buzsáki, G. (1994). The hippocampal CA3 network: An in vivo intracellular labeling study. The Journal of Comparative Neurology, 339(2), 181–208. 10.1002/cne.903390204 8300905

[brb31410-bib-0043] Long, L. L. , Bunce, J. G. , & Chrobak, J. J. (2015). Theta variation and spatiotemporal scaling along the septotemporal axis of the hippocampus. Frontiers in Systems Neuroscience, 9(March), 1–14. 10.3389/fnsys.2015.00037 25852496 PMC4360780

[brb31410-bib-0044] Loureiro, M. , Lecourtier, L. , Engeln, M. , Lopez, J. , Cosquer, B. , Geiger, K. , … Pereira de Vasconcelos, A. (2012). The ventral hippocampus is necessary for expressing a spatial memory. Brain Structure and Function, 217(1), 93–106. 10.1007/s00429-011-0332-y 21667304

[brb31410-bib-0045] Lubenov, E. V. , & Siapas, A. G. (2009). Hippocampal theta oscillations are travelling waves. Nature, 459(7246), 534–539. 10.1038/nature08010 19489117

[brb31410-bib-0046] Maren, S. , & Holt, W. G. (2004). Hippocampus and Pavlovian fear conditioning in rats: Muscimol infusions into the ventral, but not dorsal, hippocampus impair the acquisition of conditional freezing to an auditory conditional stimulus. Behavioral Neuroscience, 118(1), 97–110. 10.1037/0735-7044.118.1.97 14979786

[brb31410-bib-0047] Martin, S. J. , de Hoz, L. , & Morris, R. G. M. (2005). Retrograde amnesia: Neither partial nor complete hippocampal lesions in rats result in preferential sparing of remote spatial memory, even after reminding. Neuropsychologia, 43(4), 609–624. 10.1016/j.neuropsychologia.2004.07.007 15716151

[brb31410-bib-0048] McElroy, M. W. , & Korol, D. L. (2005). Intrahippocampal muscimol shifts learning strategy in gonadally intact young adult female rats. Learning & Memory, 12(2), 150–158. 10.1101/lm.86205 15805313 PMC1074333

[brb31410-bib-0049] McEown, K. , & Treit, D. (2010). Inactivation of the dorsal or ventral hippocampus with muscimol differentially affects fear and memory. Brain Research, 1353, 145–151. 10.1016/j.brainres.2010.07.030 20647005

[brb31410-bib-0050] Morris, R. (1984). Developments of a water‐maze procedure for studying spatial learning in the rat. Journal of Neuroscience Methods, 11(1), 47–60. 10.1016/0165-0270(84)90007-4 6471907

[brb31410-bib-0051] Moser, E. , Moser, M. B. , & Andersen, P. (1993). Spatial learning impairment parallels the magnitude of dorsal hippocampal lesions, but is hardly present following ventral lesions. The Journal of Neuroscience, 13(9), 3916–3925. 10.1523/JNEUROSCI.13-09-03916.1993 8366351 PMC6576447

[brb31410-bib-0052] Moser, M. B. , Moser, E. I. , Forrest, E. , Andersen, P. , & Morris, R. G. (1995). Spatial learning with a minislab in the dorsal hippocampus. Proceedings of the National Academy of Sciences, 92(21), 9697–9701. 10.1073/pnas.92.21.9697 PMC40869 7568200

[brb31410-bib-0053] O'Keefe, J. , & Dostrovsky, J. (1971). The hippocampus as a spatial map: Preliminary evidence from unit activity in the freely‐moving rat. Brain Research, 34, 171–175. 10.1016/0006-8993(71)90358-1 5124915

[brb31410-bib-0054] O'Keefe, J. , & Nadel, L. (1978). The hippocampus as a cognitive map. Oxford, UK: Oxford University Press.

[brb31410-bib-0055] Patel, J. , Fujisawa, S. , Berényi, A. , Royer, S. , & Buzsáki, G. (2012). Traveling theta waves along the entire septotemporal axis of the hippocampus. Neuron, 75(3), 410–417. 10.1016/j.neuron.2012.07.015 22884325 PMC3427387

[brb31410-bib-0056] Paxinos, G. , & Watson, C. (2007). The rat brain in stereotaxic coordinates, 6th ed., 170 (pp. 547–612). San Diego, CA: Elsevier Academic Press.

[brb31410-bib-0057] Pentkowski, N. S. , Blanchard, D. C. , Lever, C. , Litvin, Y. , & Blanchard, R. J. (2006). Effects of lesions to the dorsal and ventral hippocampus on defensive behaviors in rats. European Journal of Neuroscience, 23(February), 2185–2196. 10.1111/j.1460-9568.2006.04754.x 16630065

[brb31410-bib-0058] Pitkänen, A. , Pikkarainen, M. , Nurminen, N. , & Ylinen, A. (2000). Reciprocal connections between the amygdala and the hippocampal formation, perirhinal cortex, and postrhinal cortex in rat. A review. Annals of the New York Academy of Sciences, 911, 369–391. 10.1111/j.1749-6632.2000.tb06738.x 10911886

[brb31410-bib-0059] Port, R. L. , Finamore, T. L. , Noble, M. M. , & Seybold, K. S. (2000). Unilateral hippocampal damage impairs spatial cognition in rats. International Journal of Neuroscience, 103(1–4), 25–32. 10.3109/00207450009003249 10938560

[brb31410-bib-0060] Pothuizen, H. H. J. , Zhang, W. N. , Jongen‐Rêlo, A. L. , Feldon, J. , & Yee, B. K. (2004). Dissociation of function between the dorsal and the ventral hippocampus in spatial learning abilities of the rat: A within‐subject, within‐task comparison of reference and working spatial memory. European Journal of Neuroscience, 19, 705–712. 10.1111/j.0953-816X.2004.03170.x 14984421

[brb31410-bib-0061] Richmond, M. A. , Yee, B. K. , Pouzet, B. , Veenman, L. , Rawlins, J. N. , Feldon, J. , & Bannerman, D. M. (1999). Dissociating context and space within the hippocampus: Effects of complete, dorsal, and ventral excitotoxic hippocampal lesions on conditioned freezing and spatial learning. Behavioral Neuroscience, 113(6), 1189–1203. 10.1037/0735-7044.113.6.1189 10636298

[brb31410-bib-0062] Risold, P. Y. , & Swanson, L. W. (1996). Structural evidence for functional domains in the rat hippocampus. Science, 272(5267), 1484–1486. 10.1126/science.272.5267.1484 8633241

[brb31410-bib-0063] Schmidt, B. , Hinman, J. R. , Jacobson, T. K. , Szkudlarek, E. , Argraves, M. , Escabi, M. A. , & Markus, E. J. (2013). Dissociation between dorsal and ventral hippocampal theta oscillations during decision‐making. The Journal of Neuroscience, 33(14), 6212–6224. 10.1523/JNEUROSCI.2915-12.2013 23554502 PMC6618918

[brb31410-bib-0064] Shinohara, Y. , Hosoya, A. , Yahagi, K. , Ferecskó, A. S. , Yaguchi, K. , Sík, A. , … Hirase, H. (2012). Hippocampal CA3 and CA2 have distinct bilateral innervation patterns to CA1 in rodents. European Journal of Neuroscience, 35(5), 702–710. 10.1111/j.1460-9568.2012.07993.x 22339771

[brb31410-bib-0065] Suzuki, W. , & Amaral, D. (1994). Topographic organization of the reciprocal connections between the monkey entorhinal cortex and the perirhinal and parahippocampal cortices. The Journal of Neuroscience, 14(3), 1856–1877. 10.1523/JNEUROSCI.14-03-01856.1994 8126576 PMC6577578

[brb31410-bib-0066] Swanson, L. W. , Sawchenko, P. E. , & Cowan, W. M. (1980). Evidence that the commissural, associational and septal projections of the regio inferior of the hippocampus arise from the same neurons. Brain Research, 197(1), 207–212. 10.1016/0006-8993(80)90446-1 7397553

[brb31410-bib-0067] Swanson, L. W. , Wyss, J. M. , & Cowan, W. M. (1978). An autoradiographic study of the organization of intrahippocampal association pathways in the rat. The Journal of Comparative Neurology, 181, 681–716. 10.1002/cne.901720104 690280

[brb31410-bib-0068] Trent, N. L. , & Menard, J. L. (2010). The ventral hippocampus and the lateral septum work in tandem to regulate rats' open‐arm exploration in the elevated plus‐maze. Physiology and Behavior, 101(1), 141–152. 10.1016/j.physbeh.2010.04.035 20451539

[brb31410-bib-0069] Turner, D. A. , Li, X.‐G. , Pyapali, G. K. , Ylinen, A. , & Buzsaki, G. (1995). Morphometric and electrical properties of reconstructed hippocampal CA3 neurons recorded in vivo. The Journal of Comparative Neurology, 356(4), 580–594. 10.1002/cne.903560408 7560268

[brb31410-bib-0070] Van Groen, T. , & Wyss, J. M. (1990). Extrinsic projections from area CA1 of the rat hippocampus: Olfactory, cortical, subcortical, and bilateral hippocampal formation projections. The Journal of Comparative Neurology, 302(3), 515–528. 10.1002/cne.903020308 1702115

[brb31410-bib-0071] Vorhees, C. V. , & Williams, M. T. (2006). Morris water maze: Procedures for assessing spatial and related forms of learning and memory. Nature Protocols, 1(2), 848–858. 10.1038/nprot.2006.116 17406317 PMC2895266

[brb31410-bib-0072] Wang, G. W. , & Cai, J. X. (2008). Reversible disconnection of the hippocampal‐prelimbic cortical circuit impairs spatial learning but not passive avoidance learning in rats. Neurobiology of Learning and Memory, 90, 365–373. 10.1016/j.nlm.2008.05.009 18614383

[brb31410-bib-0073] Weeden, C. S. S. , Hu, N. J. , Ho, L. U. N. , & Kesner, R. P. (2014). The role of the ventral dentate gyrus in olfactory pattern separation. Hippocampus, 24(5), 553–559. 10.1002/hipo.22248 24449260

[brb31410-bib-0074] Weeden, C. S. S. , Roberts, J. M. , Kamm, A. M. , & Kesner, R. P. (2015). The role of the ventral dentate gyrus in anxiety‐based behaviors. Neurobiology of Learning and Memory, 118, 143–149. 10.1016/j.nlm.2014.12.002 25498221

[brb31410-bib-0075] Witter, M. P. (2007). Intrinsic and extrinsic wiring of CA3: Indications for connectional heterogeneity. Learning & Memory, 14(11), 705–713. 10.1101/lm.725207 18007015

[brb31410-bib-0076] Witter, M. P. , Doan, T. P. , Jacobsen, B. , Nilssen, E. S. , & Ohara, S. (2017). Architecture of the Entorhinal Cortex A Review of Entorhinal Anatomy in Rodents with Some Comparative Notes. Frontiers in Systems Neuroscience, 11(June), 1–12. 10.3389/fnsys.2017.00046 28701931 PMC5488372

[brb31410-bib-0077] Witter, M. P. , & Groenewegen, H. J. (1984). Laminar origin and septotemporal distribution of entorhinal and perirhinal projections to the hippocampus in the cat. Journal of Comparative Neurology, 224(3), 371–385. 10.1002/cne.902240305 6715585

[brb31410-bib-0078] Witter, M. P. , Naber, P. A. , van Haeften, T. , Machielsen, W. C. M. , Rombouts, S. A. R. B. , Barkhof, F. , … Lopes da Silva, F. H. (2000). Cortico‐hippocampal communication by way of parallel parahippocampal‐subicular pathways. Hippocampus, 10(4), 398–410. 10.1002/1098-1063(2000)10:4<398:AID-HIPO6>3.0.CO;2-K 10985279

[brb31410-bib-0079] Woollett, K. , & Maguire, E. A. (2011). Acquiring “the Knowledge” of London's layout drives structural brain changes. Current Biology, 21(24), 2109–2114. 10.1016/j.cub.2011.11.018 22169537 PMC3268356

[brb31410-bib-0080] Zou, L. B. , Yamada, K. , Sasa, M. , & Nabeshima, T. (1999). Two phases of behavioral plasticity in rats following unilateral excitotoxic lesion of the hippocampus. Neuroscience, 92(3), 819–826. 10.1016/S0306-4522(99)00029-9 10426524

